# Development and validation of new poisoning mortality score system for patients with acute poisoning at the emergency department

**DOI:** 10.1186/s13054-020-03408-1

**Published:** 2021-01-18

**Authors:** Kap Su Han, Su Jin Kim, Eui Jung Lee, Joong Ho Shin, Ji Sung Lee, Sung Woo Lee

**Affiliations:** 1 Department of Emergency Medicine, College of Medicine, Korea University, Goryeodae-ro 73, Seongbuk-gu, Seoul, 02841 Republic of Korea; 2 Clinical Research Center, Asan Institute for Life Sciences, Asan Medical Center, 88 Olympic-ro 43gil, Songpa-gu, Seoul, 05505 Republic of Korea

**Keywords:** Mortality, Prediction, Poisoning, Scoring system, Validation

## Abstract

**Background:**

A prediction model of mortality for patients with acute poisoning has to consider both poisoning-related characteristics and patients’ physiological conditions; moreover, it must be applicable to patients of all ages. This study aimed to develop a scoring system for predicting in-hospital mortality of patients with acute poisoning at the emergency department (ED).

**Methods:**

This was a retrospective analysis of the Injury Surveillance Cohort generated by the Korea Center for Disease Control and Prevention (KCDC) during 2011–2018. We developed the new-Poisoning Mortality Scoring system (new-PMS) to generate a prediction model using the derivation group (2011–2017 KCDC cohort). Points were computed for categories of each variable. The sum of these points was the new-PMS. The validation group (2018 KCDC cohort) was subjected to external temporal validation. The performance of new-PMS in predicting mortality was evaluated using area under the receiver operating characteristic curve (AUROC) for both the groups.

**Results:**

Of 57,326 poisoning cases, 42,568 were selected. Of these, 34,352 (80.7%) and 8216 (19.3%) were enrolled in the derivation and validation groups, respectively. The new-PMS was the sum of the points for each category of 10 predictors. The possible range of the new-PMS was 0–137 points. Hosmer–Lemeshow goodness-of-fit test showed adequate calibration for the new-PMS with *p* values of 0.093 and 0.768 in the derivation and validation groups, respectively. AUROCs of the new-PMS were 0.941 (95% CI 0.934–0.949, *p* < 0.001) and 0.946 (95% CI 0.929–0.964, *p* < 0.001) in the derivation and validation groups, respectively. The sensitivity, specificity, and accuracy of the new-PMS (cutoff value: 49 points) were 86.4%, 87.2%, and 87.2% and 85.9%, 89.5%, and 89.4% in the derivation and validation groups, respectively.

**Conclusions:**

We developed a new-PMS system based on demographic, poisoning-related variables, and vital signs observed among patients at the ED. The new-PMS showed good performance for predicting in-hospital mortality in both the derivation and validation groups. The probability of death increased according to the increase in the new-PMS. The new-PMS accurately predicted the probability of death for patients with acute poisoning. This could contribute to clinical decision making for patients with acute poisoning at the ED.

## Background

Acute poisoning is a global health problem, and prevention of mortality is essential in both intentional and accidental poisonings. Prediction of prognosis in patients with acute poisoning has clinical significance, i.e., it helps in timely and appropriate treatment. However, toxicology research lacks a well-accepted method for assessing the severity of poisoning [1–3]. The Poisoning Severity Score (PSS), which has been used in toxicology as a disease-specific scoring system, is used infrequently [4]. Further, it has been misused or modified from its original form [4]. Currently, it has limited clinical utility and is not likely to be applied to many cases owing to their unique clinical circumstances [4].

Mortality prediction in acute poisoning cases has been explored by applying various clinical scoring systems used in critical care [5, 6]. The Acute Physiology and Chronic Health Evaluation (APACHE) score and Simplified Acute Physiology Score (SAPS) are commonly applied tools in the intensive care unit; they are used for predicting the outcomes in specific poisoning cases [7, 8]. The mortality of patients depends on their physiological conditions and unique characteristics of the poisoning. The type of substance, route of exposure, and intent of poisoning affect the outcomes in patients with acute poisoning. Additionally, the toxic substances and their lethality are often unknown. A prediction model of mortality for patients with acute poisoning has to consider both poisoning-related characteristics and patients’ physiological conditions; moreover, it must be applicable to patients of all ages. The objective of this study was to develop a scoring system for predicting mortality in patients with acute poisoning at the emergency department (ED). This work will assist in treatment allocation and therapeutic decision making at early stages for patients with acute poisoning.

## Methods

### Study design and selection of study patients

This study was a retrospective analysis of a prospective cohort (from 23 EDs), namely the Injury Surveillance Cohort, which was generated by the Korea Center for Disease Control and Prevention (KCDC) from 2011 to 2018. This registry comprised of prospectively collected data on epidemiology and outcome variables of patients with injuries presented at the ED [9]. The registry included cases of poisoning as those with a type of injury. We selected patients with poisoning from this cohort. This selected registry included the baseline characteristics of patients with poisoning: age; sex; time-related factors, such as ED admission time and poison exposure time; poisoning-related variables, such as the intent of poisoning, route of exposure, type of substance (seven categories and 44 types of substances); and initial vital signs at the ED, such as systolic blood pressure (SBP), heart rate (HR), respiration rate (RR), body temperature (BT); and AVPU (A-alert, V-verbal response, P-pain response, U-unresponse) scale of mental status. The registry also contained outcome-related variables, such as mortality at ED or after hospitalization.

**Fig. 1:**
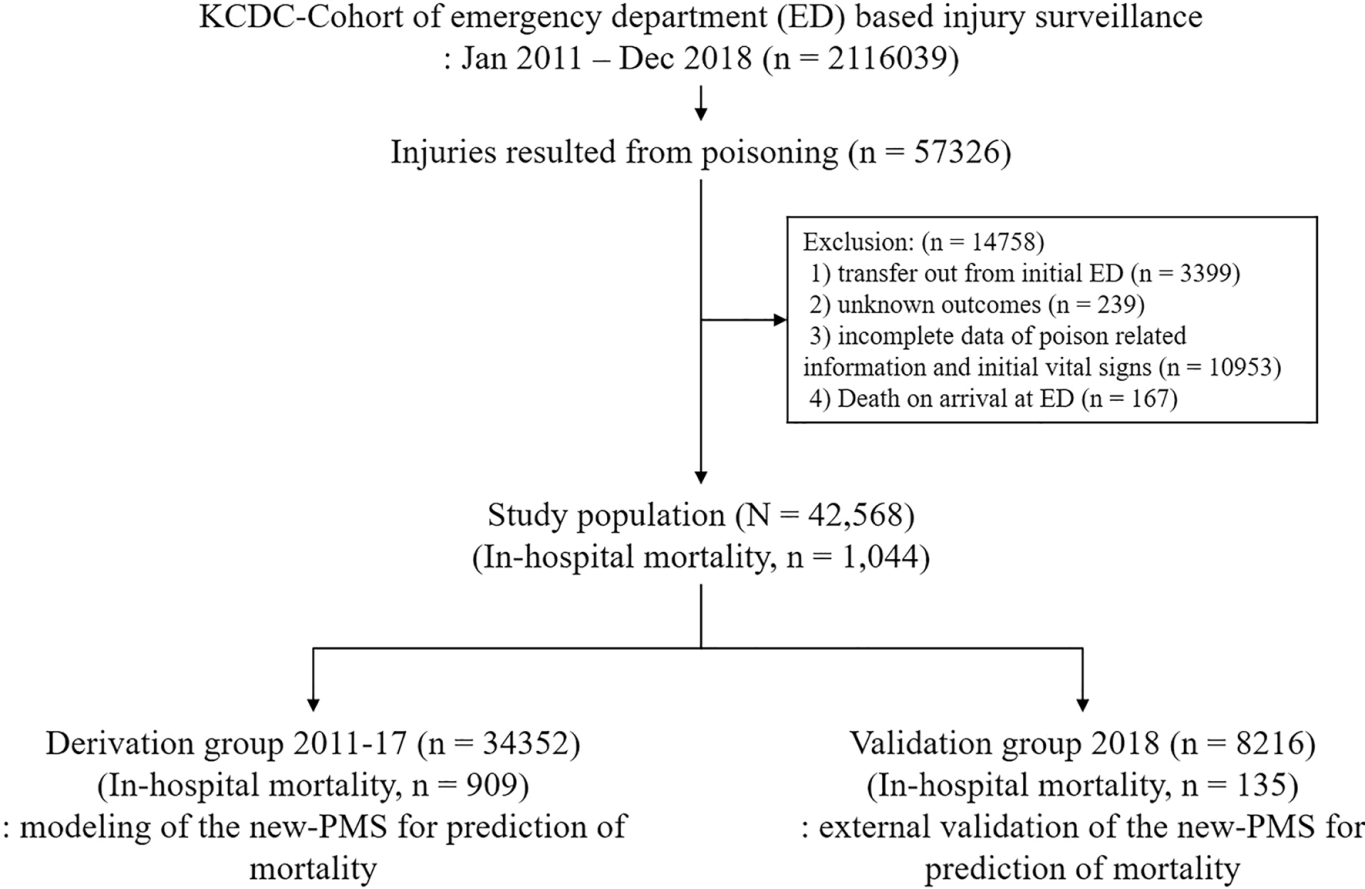
Selection of study patients. *PMS* poisoning mortality score

Patients who were transferred from the initial ED to another hospital and those who had incomplete data on poisoning-related variables, initial physiological condition-related variables or outcome-related variables, and death on arrival (DOA) at the ED were excluded from this study (Fig. 1).

The selected study population was divided into two groups: the derivation group for predicting in-hospital mortality and the validation group for external validation of the prediction model developed (Fig. 1).

The Institutional Review Board of the Korea University Hospital approved this study (IRB No. 2020AN0195).

### Data analysis

The primary outcome was in-hospital mortality. We compared the characteristics of poisoning patients between the derivation and validation groups (Table 1). Age, sex, time interval from poison exposure to ED admission, classes of substances, intent of poisoning, route of exposure, vital signs of the patients at the ED, and in-hospital mortality were analyzed (Table 1). For analysis, variables related to poisoning characteristics were categorized as follows: intent of poisoning: (1) unintentional, (2) intentional, and (3) unknown and route of exposure: (1) dermal, ocular, or contact; (2) oral; and (3) inhalation. There were 44 kinds of toxic substances that were classified into eight categories from A to H. For categorization, we considered classification of the types of substances. Further, we categorized the substances under the same classification according to the mortality index (MI) of each substance: (A) pharmaceutical agents with MI of less than 0.5%, (B) pharmaceutics with MI of 0.5–5%, (C) artificial toxic substances with MI less than 1.0%, (D) artificial toxic substances or pesticides with MI of 1.0–10.0%, (E) artificial toxic substances or pesticides with MI of 11.0–20.0%, (F) paraquat with MI of 52.5%, (G) gases with MI less than 1.0%, and (H) natural toxic substances with MI less than 1.0% (Table 2) ( Additional file 1 shows this in more detail [see Additional file 1]). MI is estimated by dividing the number of deaths by the total number of patients exposed to the specific substance. Next, it is multiplied by 100 and expressed as percentage. A patient’s physiological variables included age, SBP, HR, RR, BT, and mental status (AVPU scale). They were categorized in accordance with the predictors in SAPS-II [10]. However, SAPS-II does not include RR score. Thus, we categorized RR according to the normal range (12–24 breaths/min).

**Table 1:** Comparison of patient characteristics between the derivation and validation groups

	Derivation group (n = 34,352)	Validation group (n = 8,216)	*p* value
Demographics			
Age (years)			< 0.001
< 40, n (%)	14,432 (42.0)	3573 (43.5)	
40–59, n (%)	11,961 (34.8)	2698 (32.8)	
60–69, n (%)	3288 (9.6)	842 (10.2)	
70–74, n (%)	1592 (4.6)	275 (3.3)	
75–79, n (%)	1515 (4.4)	364 (4.4)	
≥ 80, n (%)	1564 (4.6)	464 (5.6)	
Sex			
Male: female (%)	15,514 (45.2): 18,838 (54.8)	3579 (43.6): 4637 (56.4)	0.009
Poisoning-related factors			
Time from exposure to presentation (h)	2 (1–5)	2 (1–5)	0.931
Intent of poisoning			< 0.001
Unintentional, n (%)	12,738 (37.1)	2769 (33.7)	
Intentional, n (%)	21,158 (61.6)	5421 (66.0)	
Unknown, n (%)	456 (1.3)	26 (0.3)	
Route of poisoning			< 0.001
Dermal, ocular, or contact, n (%)	346 (1.0)	4 (0.5)	
Oral ingestion, n (%)	27,531 (80.1)	6443 (78.4)	
Inhalation, n (%)	6475 (18.8)	1769 (21.1)	
Classification of substances			< 0.001
Pharmaceutics, n (%)	16,449 (47.9)	4484 (54.6)	
Pesticides, (%)	5461 (15.9)	893 (14.1)	
Gases, n (%)	6160 (17.9)	1666 (20.3)	
Artificial toxic substances, n (%)	4876 (14.2)	879 (10.7)	
Natural toxic substances, n (%)	1406 (4.1)	294 (3.6)	
Initial vital signs at emergency department			
Systolic blood pressure (mmHg)			0.081
≥ 100, n (%)	30,518 (88.8)	7349 (89.4)	
70–99, n (%)	3577 (10.4)	815 (9.9)	
≤ 69, n (%)	257 (0.7)	52 (0.6)	
Heart rate (beat/min.)			< 0.001
70–119, n (%)	27,185 (79.1)	6383 (77.7)	
30–69, n (%)	4032 (11.7)	1086 (13.2)	
120–159, n (%)	2974 (8.7)	724 (8.8)	
≥ 160, n (%)	161 (0.5)	23 (0.3)	
Respiration rate (breath/min.)			< 0.001
12–24, n (%)	31,940 (93.0)	7825 (95.2)	
≤ 11 or ≥ 25, n (%)	2412 (7.0)	391 (4.8)	
Body temperature (℃)			0.885
< 39, n (%)	34,276 (99.8)	8199 (99.8)	
≥ 39, n (%)	76 (0.2)	17 (0.2)	
Mental status, n (%)			< 0.001
Alert	24,448 (71.2)	5517 (67.1)	
Verbal response	5668 (16.5)	1628 (19.8)	
Pain response	3646 (10.6)	931 (11.3)	
Unresponse	590 (1.7)	140 (1.7)	
Outcome			
In-hospital mortality, n (%)	909 (2.6)	135 (1.6)	< 0.001

**Table 2:** Category of exposed of substances according to the class of the substance and the mortality index in the derivation group

Category	Name of substance		
A	(1) Hormones, hormone antagonists, contraceptions	(2) Diagnostic reagents	(3) Vitamin, dietary supplements
	(4) Topical preparations	(5) Acetaminophen	(6) Antipsychotics
	(7) Antidepressant	(8) Zolpidem	(9) Doxylamine
	(10) Unspecified sedatives, antipsychotics, hypnotics	(11) Benzodiazepine	
B	(1) Peptic, gastrointestinal drugs	(2) Antihistamine	(3) Cold and cough preparation
	(4) Unspecified therapeutic drugs	(5) Anticonvulsants	(6) Cardiovascular drugs
	(7) Unspecified analgesics	(8) Antibiotics, antifungals	(9) Opioid
	(10) Stimulants, street drugs	(11) Asthma therapies	(12) Oral hypoglycemic drugs
C	(1) Alcohols (liquor, ethanol, methanol)	(2) Heavy metals	(3) Hydrocarbons
	(4) Chlorine bleach, sodium hypochlorite		
D	(1) Unspecified artificial toxic substances	(2) Unspecified alkali	(3) Unspecified acid
	(4) Unspecified corrosive agents		
	(5) Rodenticide	(6) Unspecified insecticides	(7) Pyrethroid
	(8) Unspecified pesticides	(9) Unspecified herbicides	(10) Glyphosate
E	(1) Glacial acetic acid	(2) Organophosphate	(3) Carbamate
F	(1) Paraquat		
G	(1) Carbon monoxide	(2) Unspecified gases	
H	(1) Natural toxic substances		

### Development of the new poisoning mortality scoring system

We developed a new-Poisoning Mortality Scoring system (new-PMS) to generate a prediction model for the derivation group (2011–2017 data of the KCDC cohort) (Fig. 1). In the derivation group, we compared demographics, poisoning-related variables, and initial vital signs between the patients at ED who survived and were discharged (survivor subgroup) and those who died at the hospital (in-hospital death subgroup) (Table 3). We selected variables that had statistical and clinical significance in acute poisoning as predictors for developing the new-PMS [11]. Points for categories of each predictor were computed using multivariable logistic regression. The regression coefficient for each category was converted into points by dividing the smallest regression coefficient in the model (Table 4) [12]. Sum of the points for categories in the predictors was the new-PMS.

**Table 3:** Comparison of characteristics between the survivor and in-hospital death subgroups in the derivation group

	Survivor (n = 33,443)	In-hospital death (n = 909)	*p* value
Demographics			
Age (years)			
< 40, n (%)	14,373 (43.0)	59 (6.5)	< 0.001
40–59, n (%)	11,732 (35.1)	229 (25.2)	
60–69, n (%)	3139 (9.4)	149 (16.4)	
70–74, n (%)	1438 (4.3)	154 (16.9)	
75–79, n (%)	1374 (4.1)	141 (15.5)	
≥ 80, n (%)	1387 (4.1)	177 (19.5)	
Sex			
Male: female (%)	14,892(44.5): 18,551(55.5)	622(68.4): 287(31.6)	< 0.001
Poisoning-related factors			
Time from exposure to presentation (h)	2 (1–5)	2 (1–5)	0.557
Intent of poisoning			< 0.001
Unintentional, n (%)	12,629 (37.8)	109 (12.0)	
Intentional, n (%)	20,403 (61.0)	755 (83.1)	
Unknown, n (%)	411 (1.2)	45 (5.0)	
Route of poisoning			< 0.001
Dermal, ocular, or contact, n (%)	344 (0.8)	2 (0.2)	
Oral ingestion, n (%)	26,664 (79.7)	867 (95.4)	
Inhalation, n (%)	6435 (19.2)	40 (4.4)	
Category of substances			< 0.001
A, n (%)	12,609 (37.7)	41 (4.5)	
B, n (%)	3763 (11.3)	36 (4.0)	
C, n (%)	1530 (4.6)	12 (1.3)	
D, n (%)	7153 (21.4)	372 (40.9)	
E, n (%)	584 (1.7)	90 (9.9)	
F, n (%)	283 (0.8)	313 (34.4)	
G, n (%)	6123 (18.3)	37 (4.1)	
H, n (%)	1398 (4.2)	8 (0.9)	
Initial vital signs at emergency department			
Systolic blood pressure (mmHg)			< 0.001
≥ 100, n (%)	29,837 (89.2)	681 (74.9)	
70–99, n (%)	3418 (10.2)	159 (17.5)	
≤ 69, n (%)	188 (0.6)	69 (7.6)	
Heart rate (beat/min.)			< 0.001
70–119, n (%)	26,517 (79.3)	668 (73.5)	
30–69, n (%)	3913 (11.7)	119 (13.1)	
120–159, n (%)	2867 (8.6)	107 (11.8)	
≥ 160, n (%)	146 (0.4)	15 (1.7)	
Respiration rate (breath/min.)			< 0.001
12–24, n (%)	31,193 (93.3)	747 (82.2)	
≤ 11 or ≥ 25, n (%)	2250 (6.7)	162 (17.8)	
Body temperature (℃)			0.004
< 39, n (%)	33,374 (99.8)	902 (99.2)	
≥ 39, n (%)	69 (0.2)	7 (0.8)	
Mental status, n (%)			< 0.001
Alert	24,066 (72.0)	382 (42.0)	
Verbal response	5491 (16.4)	177 (19.5)	
Pain response	3420 (10.2)	226 (24.9)	
Unresponse	466 (1.4)	124 (13.6)

**Table 4:** Multivariable logistic regression for the calculation of the new-poisoning mortality scores (PMSs) for each of category of each variable in the acute poisoning patients

	B	Points = B/0.124	Odd ratio (95% confidence interval)	*p* value
Demographics				
Age (years)				
< 40, n (%)	Reference	0	1	< 0.001
40–59, n (%)	0.815	7	2.260 (1.644–3.107)	< 0.001
60–69, n (%)	1.435	12	4.198 (2.973–5.929)	< 0.001
70–74, n (%)	2.003	16	7.413 (5.201–10.566)	< 0.001
75–79, n (%)	1.955	16	7.066 (4.925–10.140)	< 0.001
≥ 80, n (%)	2.395	19	10.968 (7.737–15.548)	< 0.001
Sex				
Female	Reference	0	1	
Male	0.436	4	1.547 (1.301–1.838)	< 0.001
Poisoning-related factors				
Intent of poisoning				
Unintention	Reference	0	1	< 0.001
intention	1.039	8	2.826 (2.215–3.605)	< 0.001
Unknown	1.073	9	2.924 (1.803–4.742)	< 0.001
Route of poisoning				
Dermal, ocular, or contact	Reference	0	1	0.274
Oral	1.006	8	2.734 (0.652–11.456)	0.169
Inhalation	0.592	5	1.808 (0.332–9.835)	0.493
Category of substances				
A, n (%)	Reference	0	1	< 0.001
B, n (%)	1.373	11	3.946 (2.482–6.273)	< 0.001
C, n (%)	1.817	15	6.151 (3.159–11.978)	< 0.001
D, n (%)	2.654	21	14.213 (10.134–19.934)	< 0.001
E, n (%)	3.36	27	28.797 (19.152–43.299)	< 0.001
F, n (%)	5.866	47	352.781 (241.570–515.191)	< 0.001
G, n (%)	1.801	15	6.054 (2.199–16.670)	< 0.001
H, n (%)	1.492	12	4.444 (1.989–9.931)	< 0.001
Vital signs at emergency department				
Systolic blood pressure (mmHg)				
≥ 100	Reference	0	1	< 0.001
70–99	0.734	6	2.084 (1.654–2.627)	< 0.001
≤ 69	1.903	15	6.704 (4.563–9.849)	< 0.001
Heart rate (beats/min.)				
70–119	Reference	0	1	0.001
30–69	0.124	1	1.132 (0.885–1.447)	0.323
120–159	0.458	4	1.581 (1.197–2.087)	0.001
≥ 160	0.984	8	2.675 (1.294–5.530)	0.008
Respiration rate (breaths/min.)				
12–24	Reference	0	1	< 0.001
≤ 11 or ≥ 25	0.663	5	1.941 (1.529–2.464)	< 0.001
Body temperature (°C)				
< 39	Reference	0	1	
≥ 39	0.684	6	1.981 (0.642–6.116)	0.235
Mental status				
Alert	Reference	0	1	< 0.001
Verbal response	0.61	5	1.841 (1.474–2.300)	< 0.001
Pain response	1.017	8	2.765 (2.218–3.446)	< 0.001
Unresponse	2.033	16	7.638 (5.618–10.386)	< 0.001

Base constant B was selected as the smallest regression coefficient in the model, which was 0.124

The new-PMS was the sum of the point of each variable. The possible range of new-PMS was 0 to 137 points

### Evaluation of the new-PMS model performance

Statistical performance of the new-PMS was assessed in terms of calibration and discrimination. Hosmer–Lemeshow goodness-of-fit test was used to evaluate the agreement between observed and predicted mortalities with respect to calibration ability of the new-PMS. The discrimination performance of the new-PMS was evaluated using sensitivity, specificity, accuracy, and area under the receiver operating characteristic curve (AUROC). The optimal cutoff value for calculating sensitivity, specificity, and accuracy was the shortest distance between each point on the ROC curve and the upper left corner. External temporal validation was achieved by calculating AUROC in the validation group (2018 data of the KCDC cohort).

For simple interpretation in a clinical setting, we created risk groups. First, we created 10 score groups of equal sizes from the Hosmer–Lemeshow test. Next, we categorized them into the following four risk groups according to morality of the score groups: very low for less than 0.1%, low for 0.1–0.9%, intermediate for 1.0–9.9%, and high risk for 10.0% or higher [13]. AUROC and observed mortalities were investigated in the derivation and validation groups, respectively [11, 13]. Additionally, we introduced an equation for calculating the predicted mortality from logistic regression of the new-PMS.

### Statistical analyses

Continuous variables were reported as medians with interquartile ranges. Differences in the medians were compared using the Mann–Whitney U test. Categorical variables were compared using the Chi-square test. Sensitivity, specificity, accuracy, and AUROC were reported with 95% confidence intervals (CIs). All statistical analyses were performed using SPSS version 20.0 (IBM Corp., Armonk, NY, USA). Two-tailed *p* values less than 0.05 were considered statistically significant.

## Results

### Selection of the study population and outcomes

Of the 57,326 poisoning cases, 14,758 (25.7%) were excluded (Fig. 1). Of the 42,568 included patients, 34,352 (80.7%) and 8216 (19.3%) were enrolled in the derivation and validation groups, respectively (Fig. 1). Among the study population, the median time from poison exposure to ED presentation was 2.0 h (interquartile range 1.0–2.0 h). The incidence of in-hospital mortality was 909 (2.6%) and 135 (1.6%) for the derivation and validation groups, respectively (*p* < 0.001) (Table 1). Characteristics of the derivation and validation groups are presented in Table 1.

### Development of the new-PMS in the derivation group

We compared characteristics between the survivor and in-hospital death subgroups within the derivation group (Table 3). The demographics, poisoning-related variables, and initial vital signs of each subgroup are shown in Table 3. Patients of the in-hospital death subgroup showed higher likelihood of being older, male, undergoing intentional poisoning, oral ingestion, and pesticide poisoning; they also initially presented low SBPs, high HRs, high RRs, and altered mental statuses compared with those in the survivor subgroup. Time from poison exposure to ED presentation was not significantly different between the survivor and in-hospital death subgroups (*p* = 0.057).

We selected 10 predictors from these variables considering clinical reasoning and statistical significance. The 10 predictors (age, sex, type of substance, intent of poisoning, route of poisoning, SBP, HR, RR, BT, and AVPU scale) and categories of each predictor are presented in Table 4. Multivariable logistic regression was used to calculate the points for categories of each predictor. First, we estimated the regression coefficient (B) for categories of each predictor in the multivariable logistic regression model. Next, base constant B was selected as the smallest B in the model. The base constant B was 0.124 in the multivariable model (Table 4). We converted the B category of each predictor into points using the formula B/0.124 (Table 3) [11]. Points for each predictor are displayed in Table 4. The new-PMS was the sum of points of each predictor. The minimum-to-maximum possible range of the new-PSS was 0–137 points. Real ranges of the new-PSS were 0–117 points and 8–115 points in the derivation and validation groups, respectively.

### Performance evaluation of the new-PMS

**Fig. 2:**
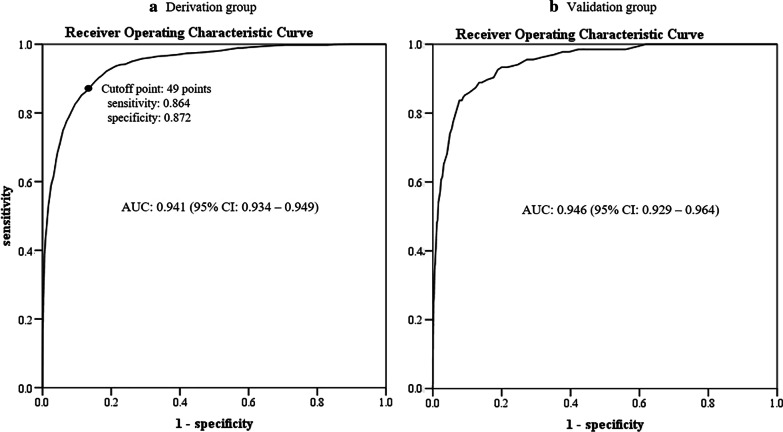
AUROCs of the new-PMS for predicting in-hospital mortality in the derivation (**a**) and validation groups (**b**). *AUROC* area under the receiver operating characteristic curve, *PMS* poisoning mortality score

The AUROC of 0.941 (95% CI: 0.934–0.949) in the derivation group was significantly high (*p* < 0.001) (Fig. 2a). The optimal cutoff value was 49 points. Statistical performance of the new-PMS in predicting in-hospital mortality for acute poisoning cases is shown in Table 5. External temporal validation analysis of the new-PMS also showed a significantly high AUROC of 0.946 (95% CI 0.929–0.964) (*p* < 0.001) in the validation group (Fig. 2b).

**Table 5:** The performance of the new-PMS for predicting mortality

Statistics	Derivation group (n = 34,352)	Validation group (n = 8216)
Hosmer–Lemeshow goodness-of-fit test	*p* = 0.093	*p* = 0.768
AUROC (95% CI)	0.941 (0.934–0.949)	0.946 (0.929–0.964)
Optimal cutoff value	49 points	49 points
Sensitivity (95% CI)	0.864 (0.841–0.886), 785/909^a^	0.859 (0.801–0.918), 116/135^a^
Specificity (95% CI)	0.872 (0.868–0.876), 29,162/33,443^a^	0.895 (0.888–0.902), 7233/8081^a^
Accuracy (95% CI)	0.872 (0.868–0.875), 29,947/34,352^a^	0.894 (0.888–0.901), 7349/8216^a^

*PMS* poisoning mortality score, *AUROC* area under receiver operating characteristic curve, *CI* confidence interval

^a^Number of patients

During risk grouping for simple interpretation in clinical settings, patients were classified according to the new-PMS into four categories: 0–27 points, very low-risk; 28–40 points, low risk; 41–55 points, intermediate risk; and 56 points or more, high risk. Table 6 presents the observed mortality according to the four risk groups. AUROCs of risk grouping were 0.920 (95% CI 0.912–0.928, *p* < 0.001) and 0.927 (95% CI 0.909–0.946, *p* < 0.001) in the derivation and validation groups, respectively. The equation for predicting in-hospital mortality was as follows: Predicted mortality = 1/(1 + e^−z^), z = − 9.763 + 0.126 × new-PMS.

**Table 6:** Risk groups within the derivation and validation groups

Risk group	New-PMS^a^	Observed mortality (%)	
		Derivation cohort (n = 34,352)	Validation cohort (n = 8216)
Very low	0–27	5/11,776 (0.04)	0/3038 (0.00)
Low	28–40	40/12,979 (0.31)	9/3266 (0.28)
Intermediate	41–55	170/6717 (2.53)	24/1322 (1.82)
High	≥ 56	694/2880 (24.10)	102/548 (18.61)

*PMS* poisoning mortality score

^a^Sum of scores for each variable as shown in Table 4

## Discussion

Outcome prediction systems for patients with poisoning are rarely studied. Thus, we developed the new-PMS to predict the probability of mortality among patients with acute poisoning. The new-PMS is a simplified scoring system that has several benefits, namely usage of objective predictors, rapid assessment of mortality risk, and early applicability in clinical settings.

Several models for severity of illness that have been used in intensive care units (ICUs) can be applied to patients with acute poisoning. Silakhori [14] reported that the APACHE-II, APACHE-IV, SAPS-II, and Sequential Organ Failure Assessment have acceptable discriminatory power for patients with poisoning, and APACHE II can be used for mortality prediction during early days of admission. However, on the first day of admission, AUROC of APACHE-II was only 0.77 in their study. They did not consider poisoning-related factors. While predicting the outcomes of acute poisoning in clinical settings, we have to consider both the physiological condition of the patient and unique characteristics of the poisoning. The new-PMS reflected two major characteristics of acute poisoning among patients, namely the characteristics of poisoning and the early physiological condition after poisoning.

Lionte [2] developed a risk-prediction nomogram for in-hospital mortality among adults poisoned with drugs and non-pharmaceutical agents. The AUROC of their nomogram was 0.949 for the validation group, which was similar to our AUROC (0.946) for the validation group. However, Lionte’s study used some test variables as predictors and included only those patients who were in the ICU or non-ICU ward, which had a small sample size. Our study was different from Lionte’s study because we performed risk grouping and introduced an equation (for clinical use) for predicting in-hospital mortality instead of using a nomogram. Further, our study included all patients with acute poisoning hospitalized, discharged from the ED with a large sample size of 23 ED-based cohort. We expect that the new-PMS can be applied for very mild to serious acute poisoning cases using the risk group and calculation of predicted mortality at the ED.

Given the unique characteristics of individual xenobiotics, many researchers have attempted to apply physiological scoring systems in patients with specific xenobiotic poisoning [7, 8, 15–18]. Peter [7] compared the performance of APACHE-II, SAPS-II, and PSS in acute organophosphate poisoning. In their study, AUROC was 0.77, 0.75, and 0.67 for APACHE-II, SAPS-II, and PSS, respectively. Previous prediction outcome models for specific toxic substances have limited value when they are applied to a wide range of patients with poisoning. In the current study, the new-PMS showed excellent performance in predicting mortality, with an AUROC of over 0.9 in all patients with acute poisoning, regardless of the cause of poisoning, type of substance, age, and sex. The present study was an attempt to develop a new scoring system (as an alternative to PSS) for outcome prediction in patients with poisoning.

We used multivariable logistic regression method to assign points for categories of each predictor. This method is commonly used for developing prognosis prediction models [11, 12]. This approach has been used in numerous studies to create a risk scoring system [19, 20]. The reference category of each predictor was determined considering the lowest mortality in SAPS II as the reference category or normal physiological variable value [10]. For example, the mortality of 40-year-olds was 0.04%, which was the lowest among all age-groups, and the point in SAPS II was 0 (Table 2).

Performance of the new-PMS was excellent according to the general guideline of AUROC in both the derivation and validation groups [21]. In simulation studies, the external validation of a prediction model requires a minimum of 100 events of the primary outcome because a small external validation study is unreliable and inaccurate [22–24]. Our validation group had 135 mortality cases from a total of 8216 poisoning cases.

For ease of use in clinical settings, we constructed four risk groups according to the new-PMS. This risk group showed a high AUROC (0.927) in the validation group. The observed mortalities increased according to the grade of risk score and showed agreement with the probability of death in the derivation and validation groups. We expect the new-PMS to be useful for objective discrimination between very-low-risk or low-risk patients, which can reduce unnecessary hospitalization. Moreover, patients with high scores can be transferred to the poisoning treatment center at early stages of treatment. Furthermore, the risk of mortality sharply increased in patients with acute poisoning with intermediate- and high-risk scores of the new-PMS. These results suggested that toxicological-specific treatment and early hemodynamic stabilization for intermediate- and high-risk patients at the ED may improve their clinical outcomes. The new PMS will contribute to clinical decision making and therapeutic guidance for patients with acute poisoning.

### Limitations

First, in this study, we excluded cases that had missing values of poisoning, outcome, and vital signs-related variables. The traditional “complete cases” analysis may lead to selection bias of subjects and statistically inefficient results [11]. Additionally, we excluded patients with DOA from this study because we considered that these patients required no specific treatments for acute poisoning. Second, the amount of exposure in cases of oral ingestion and the duration of exposure in cases of inhalation or surface absorption are important for predicting the outcomes of acute poisoning among patients. Unfortunately, our cohort did not have data on the amount of exposure and envenomation, such as animal bites. However, the new-PMS developed in this study included the unique characteristics of poisoning, such as intent and route of poisoning. Third, we categorized the toxic substances into eight categories comprising 44 specific substances. This is because all the 44 substances could not be included in the multivariable logistic regression. The clinical severity of poisoning can range from asymptomatic to lethal, depending on specificities of the toxin. For example, specific toxic substances such as paraquat are known to have high mortality irrespective of other predictors [25]. In this study, we considered paraquat as a separate category of substance. Machine learning systems have the potential to predict mortality or carry out early detection of diseases among patients in the ED [26]. Thus, we can try using several machine learning techniques with the 44 special substances without categorization for better performance of prediction models in the future. Lastly, the observed mortalities in this study were as low as 2.6% and 1.6% for the derivation and validation groups, respectively. There is a risk of overestimating/over-fitting the predictive performance of the model if the number of predictors is much larger than the number of outcome events [11].

## Conclusions

We developed a new PMS system based on demographic, poisoning-related variables, and vital signs among patients at the ED. The new-PMS showed good performance for predicting in-hospital mortality in both the derivation and validation groups, which is objective and is applicable at an early stage of poisoning. The new-PMS will contribute to clinical decision making for patients with acute poisoning at the ED.

## Supplementary Information

**Additional file 1.** Substance category according to class of the substance and mortality index.ù

## Data Availability

The datasets during and/or analyzed during the current study are available from the corresponding author upon reasonable request.
