# Supplementary material for: Development and validation of new poisoning mortality score system for patients with acute poisoning at the emergency department
Source: Crit Care. 2021 Jan 18;25:29. doi: 10.1186/s13054-020-03408-1 (PMC7814606; doi:10.1186/s13054-020-03408-1)
Supplement: Supplementary file 1 — Additional file 1. Substance category according to class of the substance and mortality index.ù [file 13054_2020_3408_MOESM1_ESM.docx]

Appendix 1. Category of exposed of substances according to the class of the substance and the mortality index in the derivation group

| Category of substances  (8 categories) | Classification  (5 classes) | Name of substance  (44 types) | No.  of exposure  (n=34352) | No.  of mortality  (n=909) | Mortality index (%)  (No. of mortality  /No. of exposure) |
| --- | --- | --- | --- | --- | --- |
| A  A  A  A  A  A  A  A  A  A  A  B  B  B  B  B  B  B  B  B  B  B  B | pharmaceutical drugs | hormones, hormone antagonists, contraceptions  diagnostic reagents  vitamin, dietary supplements  topical preparations  acetaminophen  antipsychotics  antidepressant  zolpidem  doxylamine  benzodiazepine  unspecified sedatives, antipsychotics, hypnotics  antihistamine  peptic, gastrointestinal drugs  cold and cough preparation  unspecified therapeutic drugs  anticonvulsants  cardiovascular drugs  unspecified analgesics  antibiotics, antifungals  opioid  stimulants, street drugs  asthma therapies  oral hypoglycemic drugs | 185  171  89  59  1363  419  1157  2533  349  1872  4453  204  189  371  1336  263  519  542  157  96  33  30  59 | 0  0  0  0  1  1  3  7  1  8  20  1  1  2  10  2  4  6  3  2  1  1  3 | 0.00  0.00  0.00  0.00  0.07  0.24  0.26  0.28  0.29  0.43  0.45  0.49  0.53  0.54  0.75  0.76  0.77  1.11  1.91  2.08  3.03  3.33  5.08 |
| C  C  C  C  D  D  D  D  E | artificial toxic substances | hydrocarbons  heavy metals  alcohols  chlorine bleach, sodium hypochlorite  unspecified artificial toxic substances  unspecified alkali  unspecified acid  unspecified corrosive agents  glacial acetic acid | 66  27  392  1057  2422  353  216  172  171 | 0  0  2  10  39  7  6  5  32 | 0.00  0.00  0.51  0.95  1.61  1.98  2.78  2.91  18.71 |
| D  D  D  D  D  D  E  E  F | pesticides | rodenticide  pyrethroid  unspecified insecticides  unspecified pesticides  unspecified herbicides  glyphosate  organophosphate  carbamate  paraquat | 167  275  1211  475  1361  873  393  110  596 | 2  8  64  41  120  80  45  13  313 | 1.20  2.91  5.28  8.63  8.82  9.16  11.45  11.82  52.52 |
| G  G | gases | carbon monoxide  unspecified gases | 5241  919 | 32  5 | 0.61  0.54 |
| H | natural toxic substances | natural toxic substances | 1406 | 8 | 0.57 |
